# Preclinical Positron Emission Tomography Imaging of B7–H3 Expression Using Affibody Molecules Labeled with Gallium-68

**DOI:** 10.1021/acsptsci.5c00320

**Published:** 2025-09-04

**Authors:** Vladimir Tolmachev, Ekaterina A. Bezverkhniaia, Eleftherios Papalanis, Abdullah Mujahid Bin Muhammad, Anzhelika Vorobyeva, Elin Gunneriusson, Susanne Klint, Eva Ryer, Matilda Carlqvist, Wojciech Kazmierczak, Anna Orlova, Fredrik Y. Frejd, Maryam Oroujeni

**Affiliations:** † Department of Immunology, Genetics and Pathology, 8097 Uppsala University, 751 85 Uppsala, Sweden; ‡ Department of Medicinal Chemistry, Uppsala University, 751 83 Uppsala, Sweden; § 7627 Affibody AB, 171 65 Solna, Sweden

**Keywords:** B7−H3, Affibody molecule, gallium-68 (^68^Ga), NOTA chelator, SKOV-3 xenograft, PET/CT imaging

## Abstract

Affibody molecules, nonimmunoglobulin scaffold proteins, have a high potential as probes for molecular imaging of different molecular targets. One of the molecular targets for radionuclide diagnosis and therapy is B7–H3 (known as CD276), which is overexpressed in various cancers, whereas its expression is low in most normal organs and tissues. The visualization of expression levels of B7–H3 has been performed using Affibody molecules labeled with Tc-99m. However, radionuclide molecular imaging using PET offers several advantages such as superior sensitivity, quantitation accuracy, and better spatial resolution compared to SPECT. In this study, we aimed to introduce a radiotracer for PET imaging of B7–H3. To design imaging agents for labeling with the generator-produced positron-emitting radionuclide ^68^Ga, the macrocyclic triaza chelator (2-[4,7-bis­(carboxymethyl)-1,4,7-triazonan-1-yl]­acetic acid) (NOTA) was site-specifically coupled to the C-terminal cysteine of the anti–B7-H3 Affibody molecules. Four different variants of Affibody molecules, Z_B7–H3___2_, Z_B7–H3___3_, Z_B7–H3___4_, and Z_AC12_ (as control), were produced, characterized, and successfully labeled with ^68^Ga. ^68^Ga-labeled conjugates bound specifically to B7–H3-expressing cells *in vitro* and *in vivo*. Biodistribution showed that [^68^Ga]­Ga-Z_B7–H3___2_ had the highest tumor accumulation only 2 h after administration, which was 2.8-fold higher than that for the control Z_AC12_. There was a tendency for higher tumor-to-organ ratios compared to the other variants, resulting in higher imaging contrast using [^68^Ga]­Ga-Z_B7–H3___2_ for preclinical PET imaging of B7–H3-expressing tumors. Thus, [^68^Ga]­Ga-Z_B7–H3___2_ could be a promising candidate for further development aimed at clinical PET in the future.

## Graphical abstract

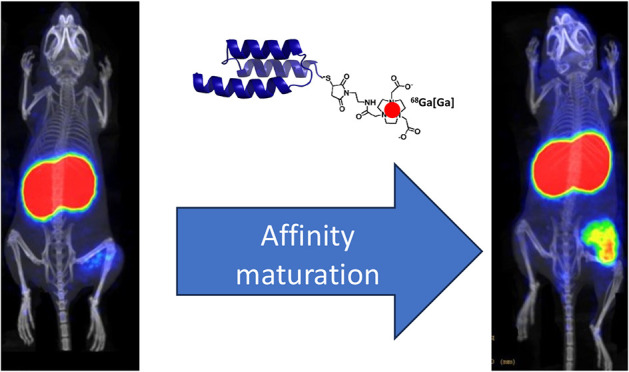

Immune checkpoint molecules are a group of cell-surface proteins that influence the immune response by providing activating or inhibitory signals with an impact on the initiation, duration, and intensity of an immune response.^1^ The immune checkpoint proteins in cancer enable malignant cells to evade immune destruction making them important therapeutic targets for immuno-oncological pharmaceuticals.^2,3^ Members of the B7 family have been identified as immune regulatory ligands that regulate T lymphocyte activation and differentiation. Among B7 family members, B7–H3 (known as CD276 or B7RP-2) is a 316 amino-acid long type I transmembrane glycoprotein encoded by a gene in 15q24 in human.^4^ The exact function of B7–H3 is still not fully understood but it has been suggested to have both immunologic and nonimmunologic functions.^1^ Growing evidence has shown that B7–H3 is aberrantly expressed in various malignancies including tumors that are less responsive to current immunotherapies. The expression is associated with progressive disease, poor patient’s survival, and poor efficacy of immune checkpoint-based treatments.^5,6^ Hence, B7–H3 has emerged as a promising therapeutic target for cancer treatment. This target is expressed at high levels in many different cancers but with a restricted expression in normal organs and tissues which makes B7–H3 a promising molecular target also for payload targeting-based approaches.^7^ Radionuclide molecular imaging can provide accurate assessment of expression of molecular targets in cancer, enabling stratification of patients for B7–H3-targeting therapies and prediction of response or resistance to certain treatments. Monoclonal antibodies (mAbs) have been used as targeting agents in radioimmunotherapy^8^ and preclinical studies^9,10^ and phase I clinical trials^11,12^ using radiolabeled antibodies have demonstrated successful radionuclide targeting of B7–H3-expressing tumors. In spite of specific tumor localization and acceptable safety in patients, the bulkiness of such agents causes a relatively slow accumulation in tumors and slow clearance of activity from blood circulation, which for imaging purposes means that several days are needed to obtain a reasonable imaging contrast. For therapeutic applications, the slow extravasation into tumors and long circulatory residence time leads to relatively high levels of radiation to the radiosensitive bone marrow.^13^ Therefore, it can be challenging to deliver a therapeutic dose to tumors without delivering unacceptably large doses to normal organs and tissues.

Reduction in the size of an imaging agent increases the tumor penetration and resulted in a faster clearance from blood and nontargeted organs. Thus, imaging contrast increases, and the optimal time between injection and imaging decreases. Importantly, the enhanced permeability and retention effect (EPR) is minimized since small proteins (less than 45 kDa) have very little accumulation in tumors by the EPR effect.^30^ Hence, the use of targeting probes smaller than monoclonal antibodies (150 kDa) has been introduced as a suitable alternative to overcome the limitations associated with mAbs. Engineered scaffold proteins (ESPs) such as Affibody molecules,^14,15^ albumin-binding domain-derived affinity proteins (ADAPTs),^16^ and designed ankyrin repeat proteins (DARPins)^17–19^ have been used for radionuclide molecular imaging of different targets. Affibody molecules, derived from a small (7 kDa) engineered scaffold protein, are promising probes for the imaging of different malignancies. Due to their small size, they have a high extravasation rate into tumors. Moreover, they are quickly cleared from blood and nontarget tissues, resulting in a reduction of time for the imaging from days to a few hours after injection and in addition an increased imaging contrast. Therefore, they are a promising alternative for molecular imaging. Their efficacy for imaging has been demonstrated in several preclinical^20–26^ and clinical^27,28^ studies for imaging of different molecular targets. The clinical data suggest that Affibody molecules are safe and well tolerated with no clinical signs of immunogenicity even after repeated monthly administration up to three years.^29^

Visualization of B7–H3 expression using molecular imaging techniques, such as single photon emission computed tomography (SPECT) and positron emission tomography (PET), can overcome several limitations associated with the use of biopsy-based methods and feasibility of PET imaging of several molecular targets in cancers using Affibody molecules has been demonstrated.^30^ SPECT-based visualization of expression levels of B7–H3 *in vivo* 2–4 h after injection was demonstrated using a ^99m^Tc- and ^111^In-labeled Affibody molecule.^31–34^ However, PET is commonly used in oncology for cancer staging, treatment planning, and monitoring response to therapy. One of the significant advantages of PET is its ability to provide quantitative information about physiological processes, allowing for the measurement of metabolic rates and receptor densities. Importantly, PET can provide superior sensitivity and quantitation accuracy compared to SPECT.^35^ Among the possible positron-emitting radioisotopes such as ^55^Co (*T*_1/2_ = 17.5 h), ^64^Cu (*T*_1/2_ = 12.7 h), and ^89^Zr (*T*_1/2_ = 78.4 h), which provide PET imaging the next day after injection, gallium-68 (^68^Ga, *T*_1/2_ = 67.6 min, β^+^ abundance 90%, E β_+max_ = 1880 keV) is a suitable positron-emitting radionuclide for same day clinical PET imaging especially for rapidly cleared imaging agents such as Affibody molecules. The good availability of ^68^Ga from a generator and the short half-life, resulting in a low absorbed dose burden to the patients are the most important features of this radionuclide. Considering these advantages, there has been significant interest in ^68^Ga-based radiopharmaceuticals, particularly for rapidly cleared peptides.^36,37^

The potential of ^68^Ga-labeled Affibody tracers for radionuclide-based diagnostics has been confirmed by the clinical evaluations using PET.^38,39^ A recently published study of the first generation of Affibody molecules demonstrated that the Affibody molecule labeled with ^68^Ga could be used for the noninvasive identification of B7–H3 expression in systemic lesions in patients with malignant tumors.^40^ However, it was concluded that the targeting properties of the Affibody-based agent should be improved to increase the tumor lesion retention time. The macrocyclic chelator NOTA is one of the best chelators for labeling with ^68^Ga, providing a high thermodynamic and kinetic stability (stability constant (log *K*_ML_) of 31).^41^ Thus, a NOTA chelator was used in this study for the labeling of Affibody molecules with ^68^Ga. This study aimed to introduce the novel Affibody-based imaging agents for the visualization of B7–H3-expressing tumors using PET. To reach the aim, maleimide derivative of the NOTA chelator was site-specifically coupled to the four variants of anti–B7-H3 Affibody molecules at the C-termini. All four variants were characterized and labeled with ^68^Ga (Figure 1). *In vitro* experiments (to test specificity and internalization) were performed using B7–H3-expressing cell lines. Biodistribution of ^68^Ga-labeled Affibody molecules was studied in mice bearing B7–H3-expressing SKOV-3 xenografts. Uptake in B7–H3-negative Ramos xenografts was used to test specificity of ^68^Ga-labeled Affibody molecules *in vivo*. PET/CT imaging was performed.

**Figure 1:**
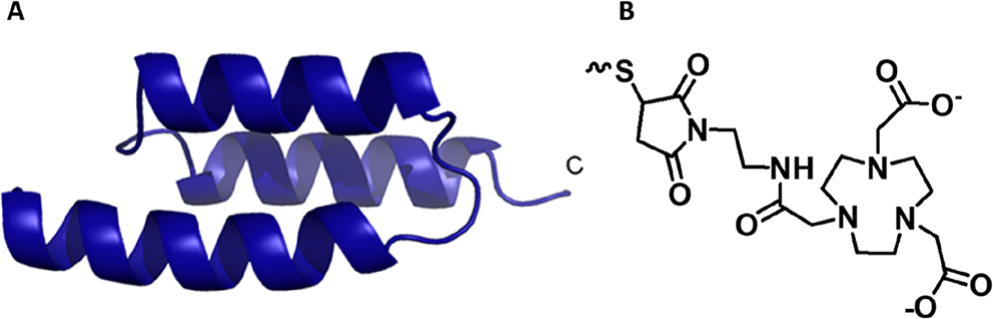
(A) Schematic structure of the tested Affibody molecules with C-terminal cysteine for the site-specific conjugation of NOTA and (B) structure of the NOTA chelator used for labeling with ^68^Ga.

## 1 Results

### 1.1 Production, Purification, and Characterization of Anti–B7-H3 Affibody Molecules

His_6_-tagged Z_B7–H3___2_, Z_B7–H3___3_, Z_B7–H3___4_ were generated based on sequences of the Z_AC12_ Affibody molecule by Stern et al.,^47^ and a previous affinity maturation by Oroujeni et al.^32^ Expressed and clarified lysates were purified using a His GraviTrap IMAC column and reverse phase chromatography (RPC). TEV protease cleavage removed the His_6_-tag and Z_B7–H3___2_, Z_B7–H3___3_, Z_B7–H3___4_, and Z_AC12_ containing a C-terminal cysteine were site-specifically conjugated with maleimide-NOTA. The identity of each Affibody molecule was confirmed using RP-UPLC-MS (Figure 2). The correct mass was confirmed for all of the Affibody molecules (Table 1).

**Figure 2:**
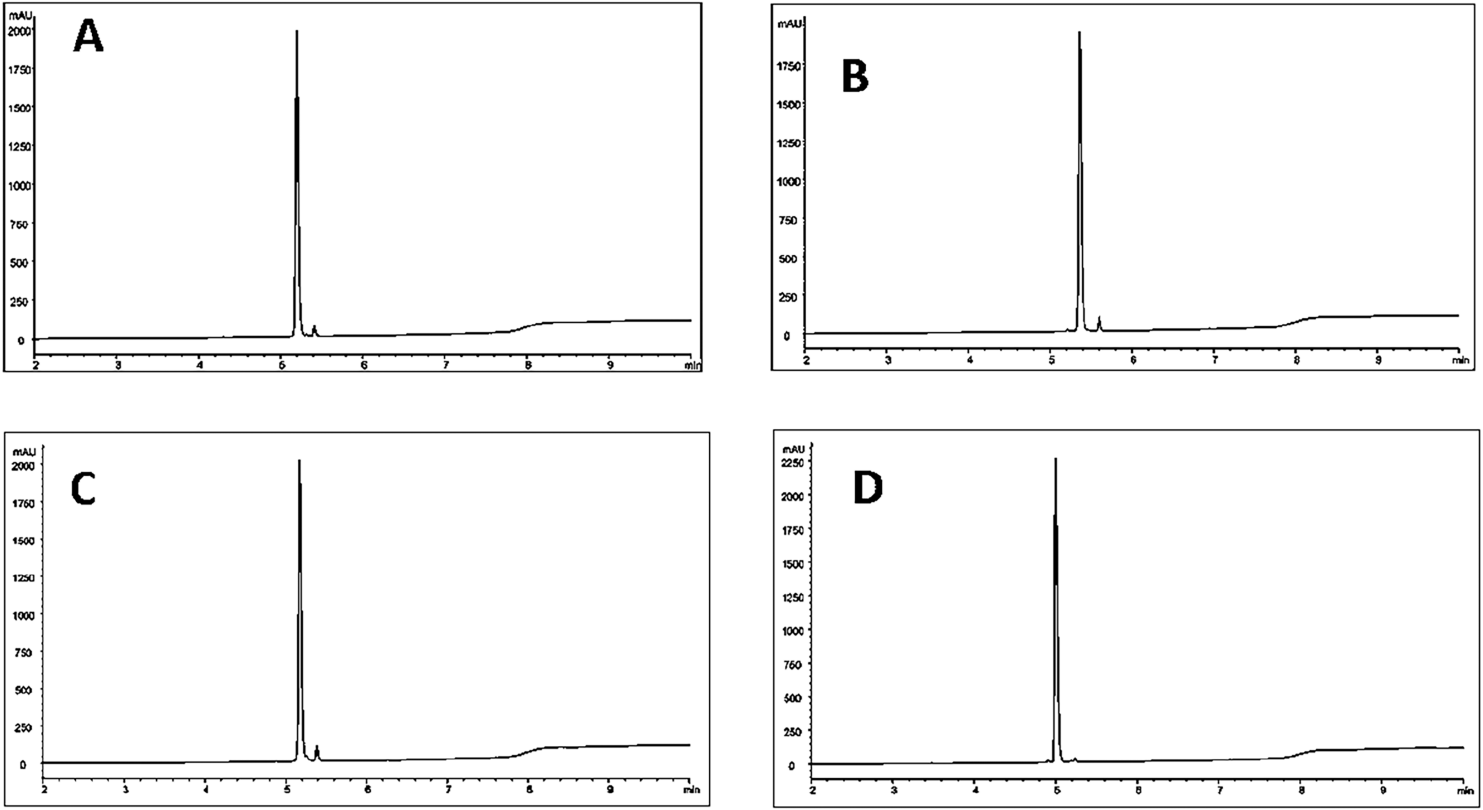
RP-UPLC chromatogram of (A) NOTA-conjugated Z_B7–H3_2_, (B) NOTA-conjugated Z_B7–H3_3_, (C) NOTA-conjugated Z_B7–H3_4_, and (D) NOTA-conjugated Z_AC12_.

**Table 1:** Results of RP-UPLC-MS Analysis of NOTA-Conjugated Affibody Molecules

	purity (%) based on A220 integration	theoretical mass (Da)	measured mass (Da)
Z_B7–H3_2_	97	7061.0	7061.55
Z_B7–H3_3_	95	7004.9	7005.39
Z_B7–H3_4_	94	7055.0	7055.52
Z_AC12_	98	6895.8	6896.32

### 1.2 CD Analysis

The CD spectra collected for His_6_-tagged Z_B7–H3_2_, Z_B7–H3_3_, and Z_B7–H3_4_ showed that all Affibody molecules have an α-helical structure at 20 °C as judged from the typical minima at 208 and 222 nm. CD spectra before and after heating to 90 °C and the melting curves are shown in Figure 3. Reversible folding was seen for all variants when spectra measured before and after heating to 90 °C were superimposed. The melting temperatures (Tm) were determined to 68 °C for Z_B7–H3_2_, 54 °C for Z_B7–H3_3_, and 58 °C for Z_B7–H3_4_.

**Figure 3:**
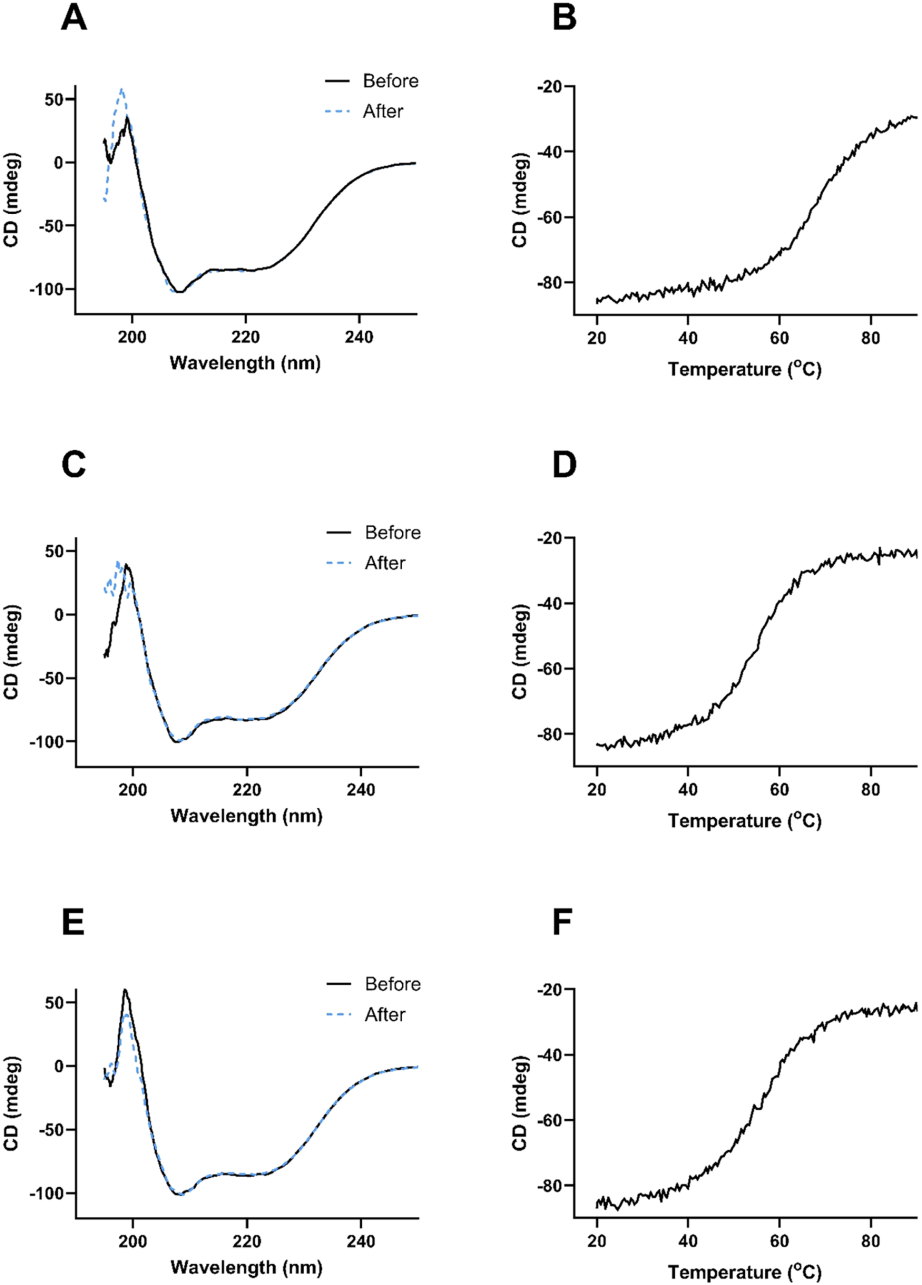
CD spectra collected at 20 °C before (solid black line) and after (broken blue line) heat-induced denaturation of (A) Z_B7–H3___2_, (C) Z_B7–H3___3_, and (E) Z_B7–H3___4_ as well as melting curves of (B) Z_B7–H3___2_, (D) Z_B7–H3___3_, and (F) Z_B7–H3___4_.

### 1.3 Binding Study Using SPR

The binding of NOTA-conjugated Z_B7–H3___2_, Z_B7–H3___3_, Z_B7–H3___4_, and Z_AC12_ to human B7–H3­(4Ig) was confirmed. The apparent binding was shown to be approximately the same for the affinity matured Affibody molecules Z_B7–H3___2_, Z_B7–H3___3_, and Z_B7–H3___4_ but improved over reference binder Z_AC12_ (Figure 4)

**Figure 4:**
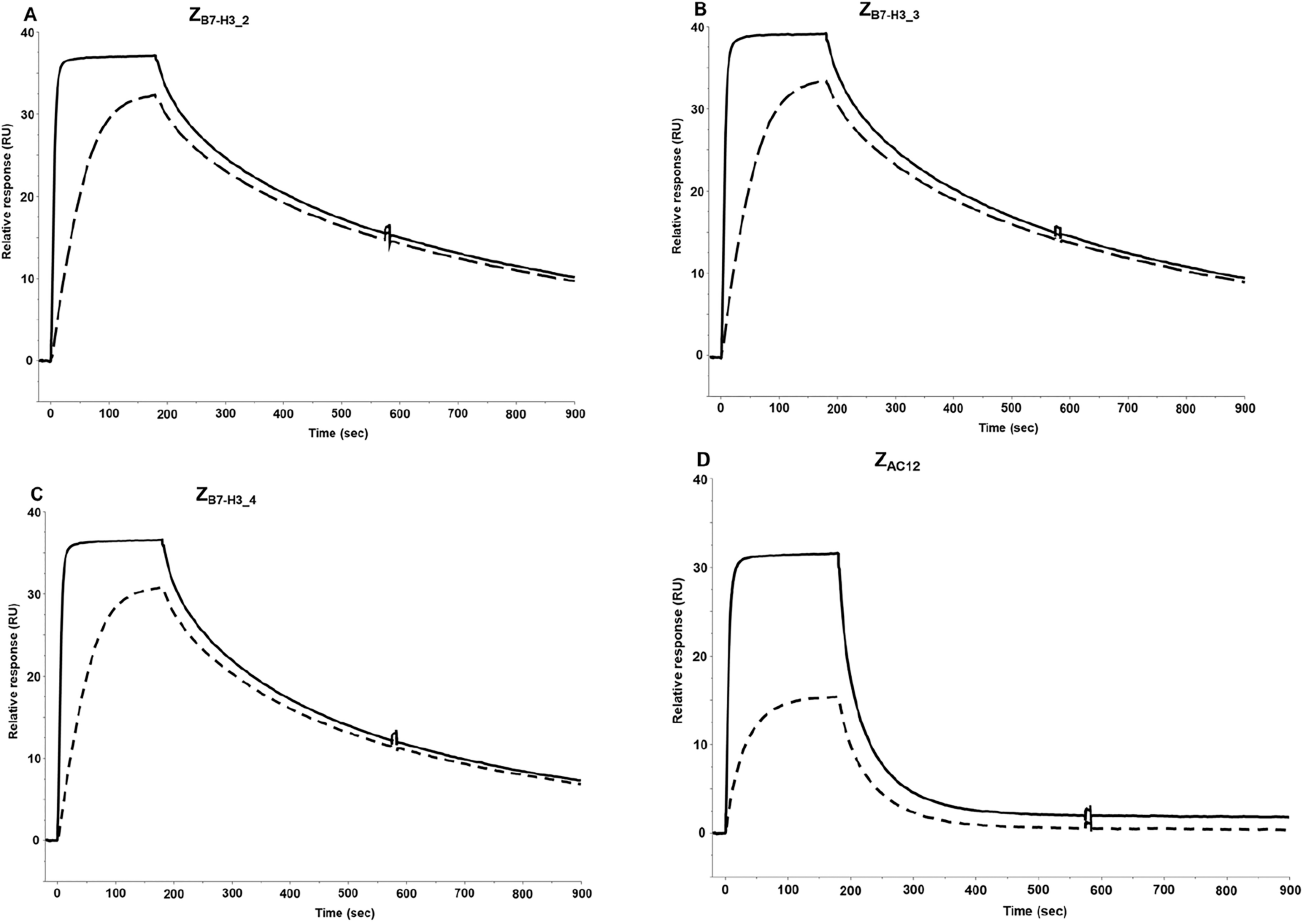
Binding of Z_B7–H3_2_, Z_B7–H3_3_, Z_B7–H3_4_, and Z_AC12_ to human B7–H3­(4Ig), as determined by SPR (Biacore). Human B7–H3­(4Ig) was immobilized on the CM5 chip followed by injections of 5 nM (dashed line) and 50 nM (solid line) Z_B7–H3_2_ (A), Z_B7–H3_3_ (B), Z_B7–H3_4_ (C), and Z_AC12_ (D). The apparent binding was shown to be approximately the same for Z_B7–H3_2_, Z_B7–H3_3_, and Z_B7–H3_4_ but improved over reference binder Z_AC12_.

### 1.4 Binding to B7–H3-Expressing Cells

Z_B7–H3_2_, Z_B7–H3_3_, Z_B7–H3_4_, and Z_AC12_ were tested in terms of binding to B7–H3-expressing SKOV-3 cells. Binding curves were plotted and are shown in Figure 5. EC50 values were determined by using the software GraphPad Prism and are shown in Table 2. The data suggest that the affinity matured Affibody molecules Z_B7–H3_2_, Z_B7–H3_3_, Z_B7–H3_4_ had 6–8 times better binding capacity to SKOV-3 cells than Z_AC12_.

**Figure 5:**
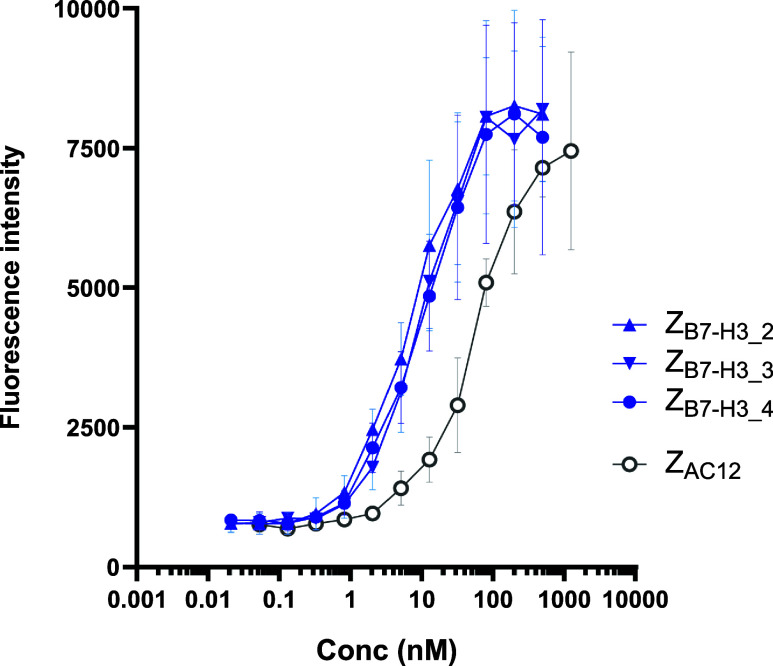
Binding of Affibody molecules to B7–H3-expressing SKOV-3 cells.

**Table 2:** EC50 Determination from SKOV-3 Binding

	Z_B7–H3__2	Z_B7–H3_3_	Z_B7–H3_4_	Z_AC12_
EC_50_ (nM)	7.3	9.9	10.1	57.1

### 1.5 Radiolabeling and *In Vitro* Stability

All variants were successfully labeled with ^68^Ga (Table 3). The radio conjugates were purified using size exclusion chromatography with NAP-5 columns providing radiochemical purities over 98%. They demonstrated high stability during incubation with a 1000-fold molar excess of EDTA and PBS for up to 2 h (Table 4). Less than 6% release of ^68^Ga from the radio conjugates was observed.

**Table 3:** Radiolabeling of Affibody molecules with ^68^Ga

	radiochemical yield, %	radiochemical purity, %	maximal specific activity, MBq/μg
Z_B7–H3___2_	89.7 ± 1.8	99.9 ± 0.2	1.2
Z_B7–H3___3_	87.6 ± 1.6	99.8 ± 0.4	1.2
Z_B7–H3___4_	90 ± 2	99.7 ± 0.6	1.2
Z_AC12_	89.6 ± 1.5	99.9 ± 0.1	1.2

**Table 4:** *In Vitro* Stability of ^68^Ga-Labeled Affibody Molecules^a^

	protein-associated activity, %	
	2 h	
	PBS	1000× EDTA
Z_B7–H3___2_	96.8 ± 0.3	95.6 ± 0.4
Z_B7–H3___3_	95.6 ± 0.7	95.8 ± 0.1
Z_B7–H3___4_	94.0 ± 0.6	94.4 ± 0.2
Z_AC12_	95.8 ± 0.1	95.1 ± 0.3

a The stability test was performed in triplicates.

According to radio-HPLC radiochromatogram (Figure 6), the retention time of ^68^Ga-labeled conjugates was around 10–12 min. Only one major peak was observed, corresponding to radiolabeled compound.

**Figure 6:**
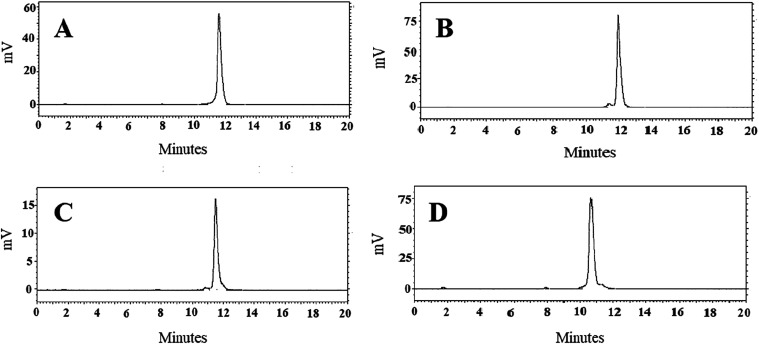
Reversed-phase HPLC radiochromatogram of (A) [^68^Ga]­Ga-Z_B7–H3___2_, (B) [^68^Ga]­Ga-Z_B7–H3___3_, (C) [^68^Ga]­Ga-Z_B7–H3___4_, and (D) [^68^Ga]­Ga-Z_AC12_ Affibody molecules. The retention times (*R_t_*) are expressed in minutes.

### 1.6 *In Vitro* Studies

The cell-associated bound activity was significantly (*p* < 0.05) decreased when the cells were presaturated with an excess amount of the nonlabeled anti–B7-H3 Affibody molecule (Figure 7) before adding the radiolabeled one, demonstrating that the binding was B7–H3-mediated *in vitro*.

**Figure 7:**
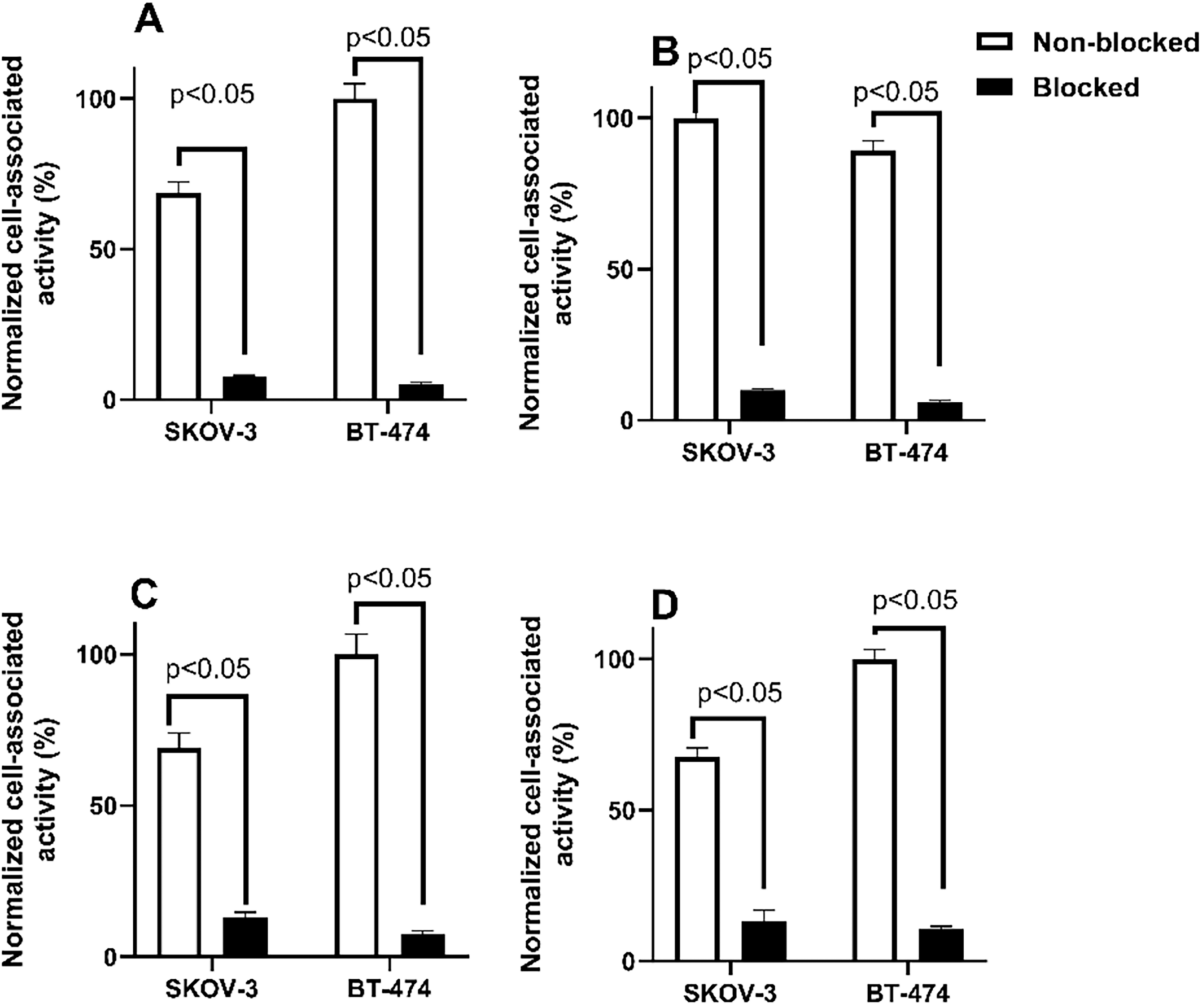
*In vitro* binding specificity of (A) [^68^Ga]­Ga-Z_B7–H3___2_, (B) [^68^Ga]­Ga-Z_B7–H3___3_, (C) [^68^Ga]­Ga-Z_B7–H3___4_, and (D) [^68^Ga]­Ga-Z_AC12_ Affibody molecules on SKOV-3 and BT-474 cells. The data are presented as an average value from three samples ± SD. Data are normalized to the average value of cell-associated radioactivity for the highest value.

The cellular processing and internalization (Figure 8) showed that total cell-associated bound activity was 3.8 ± 0.11, 4.01 ± 0.20, 3.76 ± 0.21, and 1.44 ± 0.04% on SKOV-3 for [^68^Ga]­Ga-Z_B7–H3___2_, [^68^Ga]­Ga-Z_B7–H3___3_, [^68^Ga]­Ga-Z_B7–H3___4_, and [^68^Ga]­Ga-Z_AC12_ Affibody molecules at 3 h incubation, respectively. The pattern on BT-474 was similar to SKOV-3 (4.6 ± 0.1, 4.03 ± 0.18, 3.69 ± 0.07, and 1.12 ± 0.13%, respectively). The overall internalization pattern was similar for all radio conjugates on both cell lines with a slow internalization rate.

**Figure 8:**
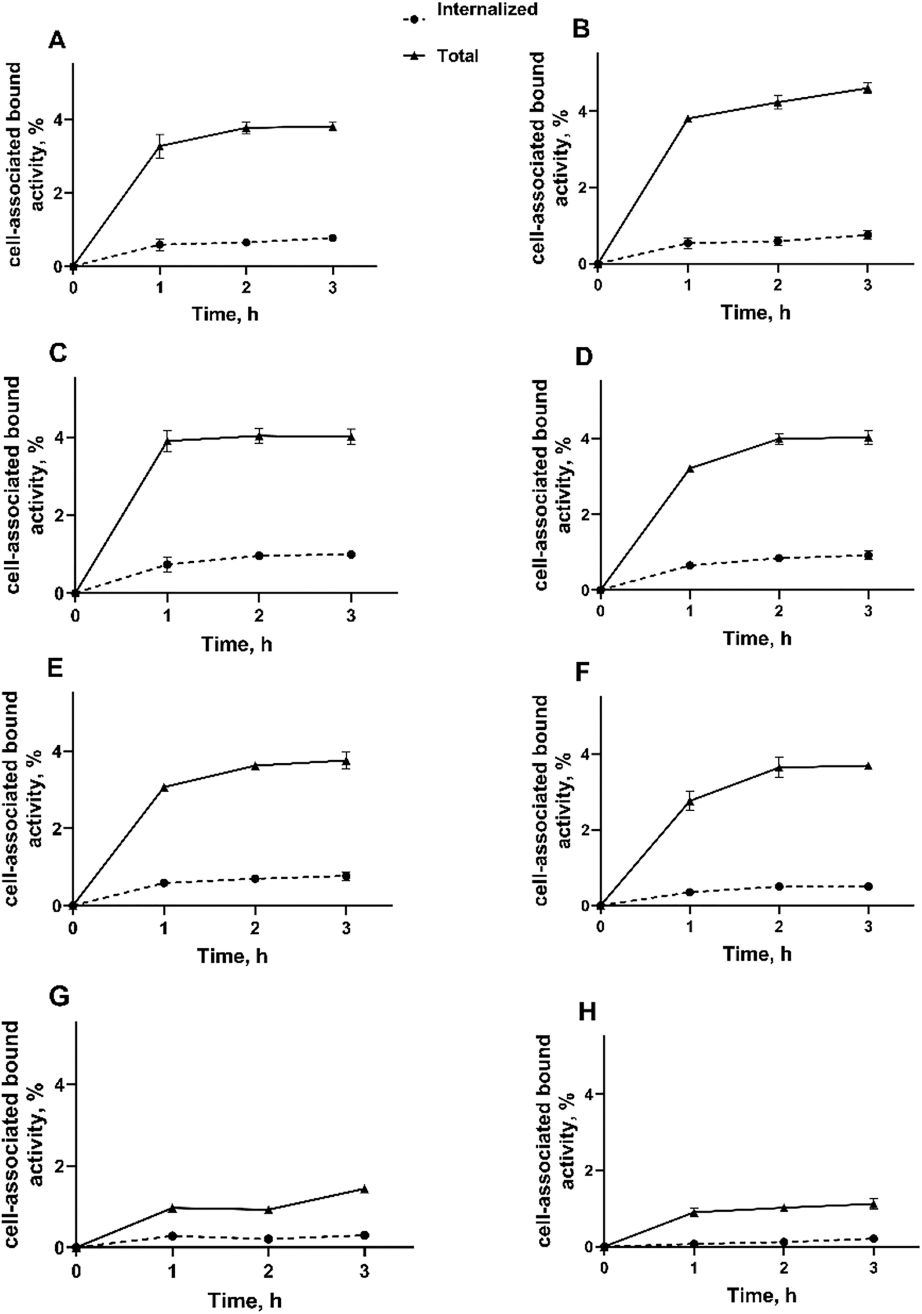
Cellular processing of (A, B) [^68^Ga]­Ga-Z_B7–H3___2_, (C, D) [^68^Ga]­Ga-Z_B7–H3___3_, (E, F) [^68^Ga]­Ga-Z_B7–H3___4_, and (G, H) [^68^Ga]­Ga-Z_AC12_ on SKOV3 (A, C, E, G) and BT-474 (B, D, F, H) cells. The data are presented as an average (*n* = 3) and SD. Error bars might not be visible, as they are smaller than the symbols.

### 1.7 *In Vivo* Studies

Biodistribution of ^68^Ga-labeled Affibody molecules in nude mice bearing human cancer xenografts demonstrated that the uptake of both ^68^Ga-labeled conjugates in B7–H3-positive SKOV-3 xenografts was significantly (*p* < 0.05) higher than in B7–H3-negative Ramos xenografts 2 h after injection (Figure 9), demonstrating *in vivo* B7–H3-specific accumulation in tumors.

**Figure 9:**
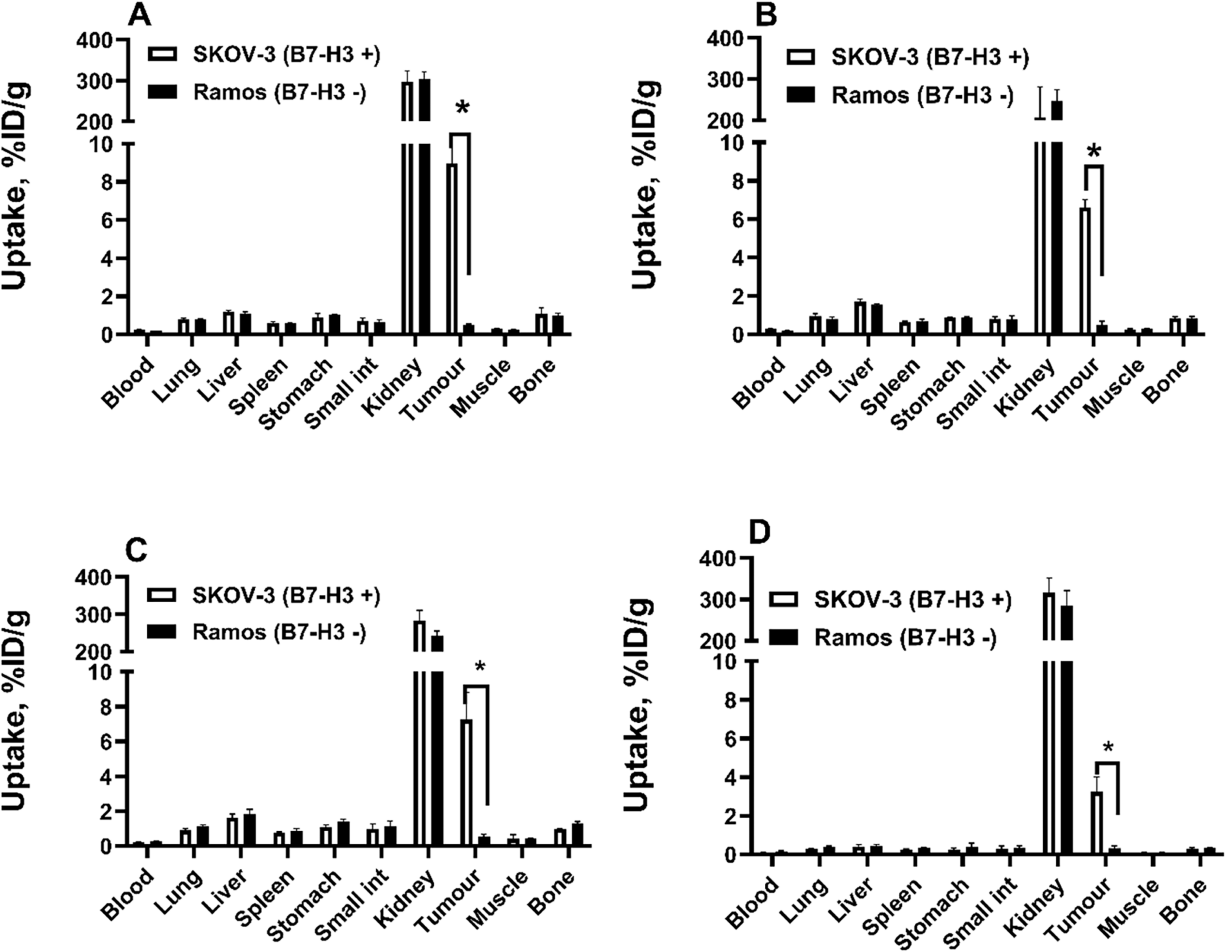
Uptake of (A) [^68^Ga]­Ga-Z_B7–H3___2_, (B) [^68^Ga]­Ga-Z_B7–H3___3_, (C) [^68^Ga]­Ga-Z_B7–H3___4_, and (D) [^68^Ga]­Ga-Z_AC12_ Affibody molecules in SKOV-3 (B7–H3-positive) and Ramos (B7–H3-negative) xenografts at 2 h after injection. Data are expressed as %ID/g and are averages from four mice ± SD. *P*-value was obtained in an unpaired *t* test.

The results of biodistribution of anti–B7-H3 Affibody molecules labeled with ^68^Ga in tumor-bearing mice 2 h after injection are presented in Table 5. Biodistribution demonstrated that the blood concentration of [^68^Ga]­Ga-Z_B7–H3___2_ (0.24 ± 0.03%ID/g) and [^68^Ga]­Ga-Z_B7–H3___3_ (0.27 ± 0.02%ID/g) was significantly (*p* < 0.05) higher than for the control variant, [^68^Ga]­Ga-Z_AC12_ (0.11 ± 0.01%ID/g), 2 h after injection. Uptake in almost all organs and tissues followed the blood concentration. For example, the uptake in lung, spleen, and stomach was significantly (*p* < 0.05) lower for control variant, [^68^Ga]­Ga-Z_AC12_, compared to the new variants. Tumor uptake of all three new variants, [^68^Ga]­Ga-Z_B7–H3___2_ (8.96 ± 1.16%ID/g), [^68^Ga]­Ga-Z_B7–H3___3_ (6.61 ± 0.41%ID/g), and [^68^Ga]­Ga-Z_B7–H3___4_ (7.25 ± 1.56^%^ID/g) was significantly (*p* < 0.05) higher than for the control variant, [^68^Ga]­Ga-Z_AC12_ (3.24 ± 0.77%ID/g). [^68^Ga]­Ga-Z_AC12_ showed the significantly (*p* < 0.05) lower hepatic uptake (0.41 ± 0.09%ID/g) compared to [^68^Ga]­Ga-Z_B7–H3___2_ (1.19 ± 0.07%ID/g), [^68^Ga]­Ga-Z_B7–H3___3_ (1.69 ± 0.14%ID/g), and [^68^Ga]­Ga-Z_B7–H3___4_ (1.63 ± 0.21%ID/g), respectively.

**Table 5:** Comparative Biodistribution of ^68^Ga-Labeled Affibody Molecules in BALB/C nu/nu Mice Bearing SKOV-3 Xenografts 2 h after Injection^a^,^h^

site	[^68^Ga]Ga-Z_B7–H3___2_	[^68^Ga]Ga-Z_B7–H3___3_	[^68^Ga]Ga-Z_B7–H3___4_	[^68^Ga]Ga-Z_AC12_
blood	0.24 ± 0.03^d^	0.27 ± 0.02^f^	0.21 ± 0.04^g^	0.11 ± 0.01^d^ ^,^ ^f^ ^,^ ^g^
lung	0.78 ± 0.07^d^	0.96 ± 0.11^f^	0.90 ± 0.11^g^	0.28 ± 0.04^d^ ^,^ ^f^ ^,^ ^g^
liver	1.19 ± 0.07^b^ ^,^ ^c^ ^,^ ^d^	1.69 ± 0.14^b^ ^,^ ^f^	1.63 ± 0.21^c^ ^,^ ^g^	0.41 ± 0.09^d^ ^,^ ^f^ ^,^ ^g^
spleen	0.59 ± 0.07^c^ ^,^ ^d^	0.64 ± 0.05^e^ ^,^ ^f^	0.78 ± 0.04^c^ ^,^ ^e^ ^,^ ^g^	0.25 ± 0.04^d^ ^,^ ^f^ ^,^ ^g^
stomach	0.90 ± 0.19^d^	0.87 ± 0.04^f^	1.09 ± 0.12^g^	0.26 ± 0.07^d^ ^,^ ^f^ ^,^ ^g^
small int	0.71 ± 0.15	0.80 ± 0.12^f^	0.96 ± 0.30^g^	0.31 ± 0.13^f^ ^,^ ^g^
kidney	296.93 ± 26.87	207.45 ± 73.64^f^	282.93 ± 27.34	315.76 ± 35.56^f^
tumor	8.96 ± 1.16^d^	6.61 ± 0.41^f^	7.25 ± 1.56^g^	3.24 ± 0.77^d^ ^,^ ^f^ ^,^ ^g^
muscle	0.27 ± 0.04	0.26 ± 0.03	0.41 ± 0.23^g^	0.08 ± 0.02^g^
bone	1.07 ± 0.32^d^	0.83 ± 0.09^f^	0.96 ± 0.05^g^	0.30 ± 0.06^d^ ^,^ ^f^ ^,^ ^g^
low GI tract**	1.07 ± 0.33	1.28 ± 0.44	1.33 ± 0.31	0.53 ± 0.24
carcass**	9.46 ± 1.65	8.30 ± 0.55	9.39 ± 1.49	2.41 ± 0.73

a Data are expressed as the percentage of administered activity (injected probe) per gram of tissue (% ID/g). The data are presented as the average value (*n* = 4) ± SD.

b Significant difference (*p* < 0.05) between [^68^Ga]­Ga-Z_B7–H3___2_ and [^68^Ga]­Ga-Z_B7–H3___3_;

c Significant difference (*p* < 0.05) between [^68^Ga]­Ga-Z_B7–H3___2_ and [^68^Ga]­Ga-Z_B7–H3___4_;

d Significant difference (*p* < 0.05) between [^68^Ga]­Ga-Z_B7–H3___2_ and [^68^Ga]­Ga-Z_AC12_;

e Significant difference (*p* < 0.05) between [^68^Ga]­Ga-Z_B7–H3___3_ and [^68^Ga]­Ga-Z_B7–H3___4_;

f Significant difference (*p* < 0.05) between [^68^Ga]­Ga-Z_B7–H3___3_ and [^68^Ga]­Ga-Z_AC12_;

g Significant difference (*p* < 0.05) between [^68^Ga]­Ga-Z_B7–H3___4_ and [^68^Ga]­Ga-Z_AC12_.

h ANOVA test (Bonferroni’s multiple comparisons test) was performed to test significant (*p* < 0.05) difference. ** Data for gastrointestinal (GI) tract with content and carcass are presented as % of injected dose per whole sample.

The results of tumor-to-organ ratios of ^68^Ga-labeled Affibody molecules in tumor-bearing mice 2 h after injection are presented in Table 6. No significant difference in the tumor-to-blood ratio was observed between each pair. However, there is a tendency of higher tumor-to-blood ratio for [^68^Ga]­Ga-Z_B7-H3_2_. Tumor-to-liver ratio was significantly (*p* < 0.05) higher for **[** ^68^Ga]­Ga-Z_B7–H3___2_ (7.5 ± 0.7) than for [^68^Ga]­Ga-Z_B7–H3___3_ (3.9 ± 0.2) and [^68^Ga]­Ga-Z_B7–H3___4_ (4.4 ± 0.8). Tumor-to-lung ratio was significantly (*p* < 0.05) higher for [^68^Ga]Ga Z_B7–H3___2_ (11.6 ± 1.2) than for [^68^Ga]­Ga-Z_B7–H3___4_ (8.0 ± 1.5).

**Table 6:** Tumor-To-Organ Ratios of ^68^Ga-Labeled Affibody Molecules in BALB/C nu/nu Mice Bearing SKOV-3 Xenografts 2 h after Injection^a^,^d^,^e^,^h^

site	[^68^Ga] Ga-Z_B7–H3___2_	[^68^Ga] Ga-Z_B7–H3___3_	[^68^Ga] Ga-Z_B7–H3___4_	[^68^Ga] Ga-Z_AC12_
blood	37.3 ± 5.6	24.7 ± 1.4	35.3 ± 8.5	28.0 ± 4.8
lung	11.6 ± 1.2^b^ ^,^ ^c^	6.9 ± 0.7^b^ ^,^ ^f^	8.0 ± 1.5^c^ ^,^ ^g^	11.6 ± 1.3^f^ ^,^ ^g^
liver	7.5 ± 0.7^b^ ^,^ ^c^	3.9 ± 0.2^b^ ^,^ ^f^	4.4 ± 0.8^c^ ^,^ ^g^	7.9 ± 1.0^f^,^g^
spleen	15.3 ± 1.2^b^ ^,^ ^c^	10.4 ± 1.0^b^	9.2 ± 1.8^c^ ^,^ ^g^	13.2 ± 2.1^g^
stomach	10.2 ± 1.6	7.6 ± 0.7^f^	6.6 ± 1.2^g^	13.2 ± 4.1^f^ ^,^ ^g^
small int	13.1 ± 3.5^c^	8.4 ± 1.1	8.0 ± 2.3^c^	11.5 ± 3.2
kidney	0.030 ± 0.003	0.036 ± 0.015	0.025 ± 0.004	0.010 ± 0.002
muscle	34.5 ± 7.8	25.5 ± 3.7	25.2 ± 9.5^g^	40.7 ± 8.4^g^
bone	8.7 ± 1.8	8.1 ± 1.3	7.6 ± 1.7	11.1 ± 2.0

a The data are presented as the average (*n* = 4) and SD.

b Significant difference (*p* < 0.05) between [^68^Ga]­Ga-Z_B7–H3___2_ and [^68^Ga]­Ga-Z_B7–H3___3_;

c significant difference (*p* < 0.05) between [^68^Ga]­Ga-Z_B7–H3___2_ and [^68^Ga]­Ga-Z_B7–H3___4_;

d significant difference (*p* < 0.05) between [^68^Ga]­Ga-Z_B7–H3___2_ and [^68^Ga]­Ga-Z_AC12_;

e significant difference (*p* < 0.05) between [^68^Ga]­Ga-Z_B7–H3___3_ and [^68^Ga]­Ga-Z_B7–H3___4_;

f significant difference (*p* < 0.05) between [^68^Ga]­Ga-Z_B7–H3___3_ and [^68^Ga]­Ga-Z_AC12_;

g significant difference (*p* < 0.05) between [^68^Ga]­Ga- Z_B7–H3___4_ and [^68^Ga]­Ga-Z_AC12_.

h ANOVA test (Bonferroni’s multiple comparisons test) was performed to test significant (*p* < 0.05) difference.

Results of nanoPET/CT imaging (Figure 10) of the ^68^Ga-labeled Affibody molecule in Balb/c nu/nu mice bearing B7–H3-positive SKOV-3 xenografts 2 h after injection confirmed ex vivo biodistribution data. A pronouncedly higher accumulation of activity in the tumor for [^68^Ga]Ga Z_B7–H3___2_ compared to other variants was observed. Activity uptake in B7–H3-negative Ramos xenografts was appreciably lower than in the B7–H3-positive SKOV-3 xenograft, which confirmed B7–H3-mediated binding of [^68^Ga]­Ga-Z_B7–H3___2_ *in vivo* (Figure 11).

**Figure 10:**
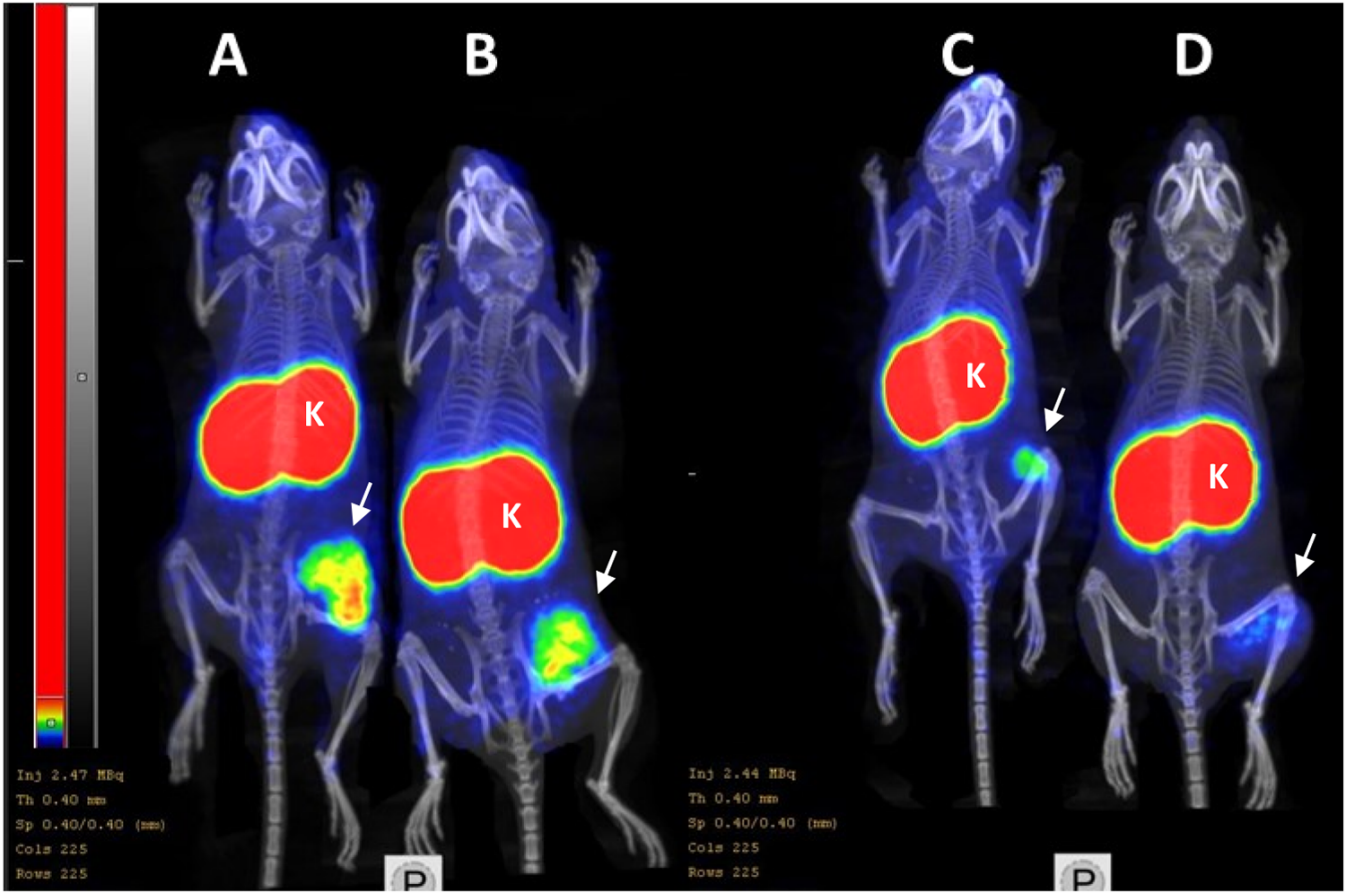
Imaging of (A) [^68^Ga]­Ga-Z_B7–H3___2_, (B) [^68^Ga]­Ga-Z_B7–H3___3_, (C) [^68^Ga]­Ga-Z_B7–H3___4_, and (D) [^68^Ga]­Ga-Z_AC12_ in BALB/C nu/nu mice bearing B7–H3-positive SKOV-3 xenografts 2 h after injection. Arrows indicate tumors, and K marks kidneys.

**Figure 11:**
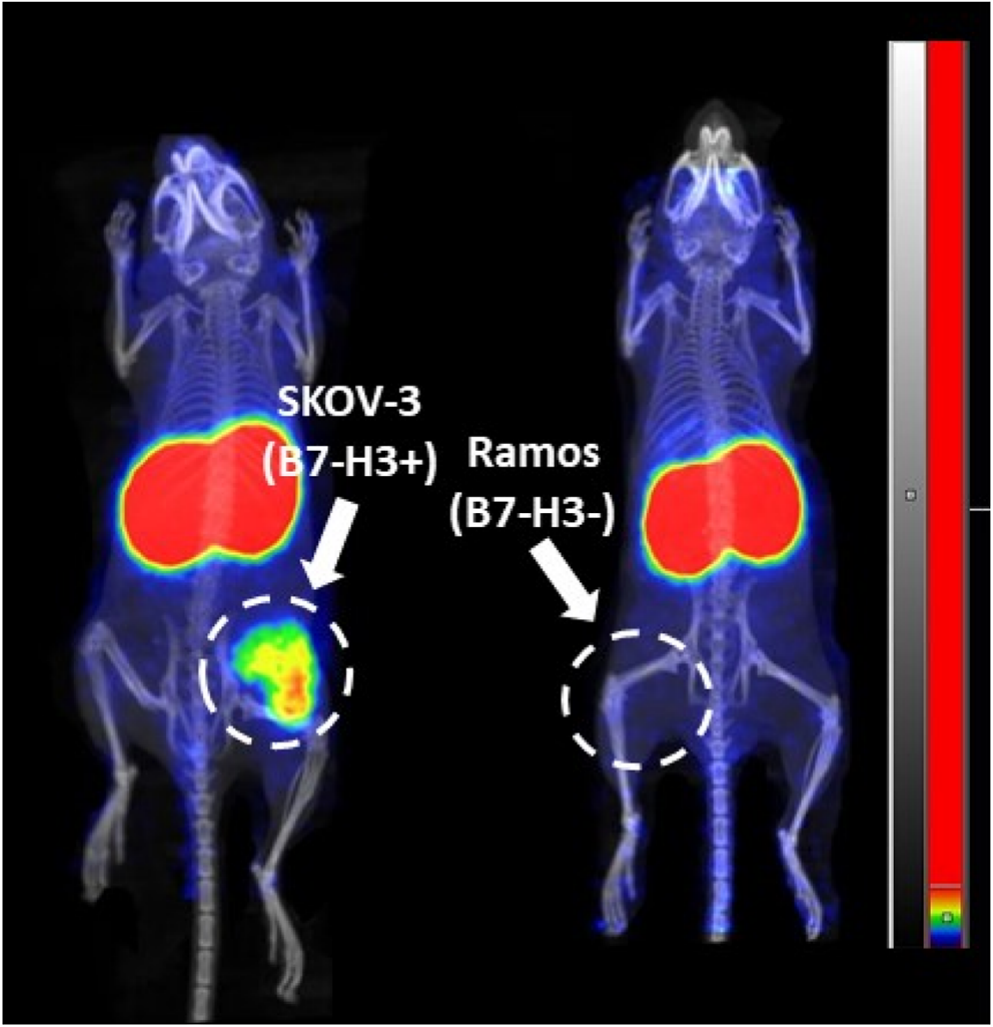
Imaging of [^68^Ga]­Ga-Z_B7–H3___2_ in BALB/C nu/nu mice bearing B7–H3-negative Ramos xenografts and B7–H3-positive SKOV-3 at 2 h after injection. Arrows point at tumors (T). Kidneys are seen in red.

## 2 Discussion

High sensitivity provided by an imaging agent or imaging modality is an essential precondition for successful translation of the radionuclide-based imaging agent into clinical use. The sensitivity depends on imaging contrast (tumor-to-organ ratios). Thus, to develop new imaging agents, the focus should be on factors increasing the imaging contrast. The use of small scaffold proteins, such as Affibody molecules, offers an improved imaging contrast compared with the use of monoclonal antibodies in radionuclide molecular imaging due to better tumor extravasation and faster blood clearance.^15^ Thus, high-contrast imaging is achieved on the same day as the injection, facilitating clinical routine use. Combination of the small size of the imaging agent with the use of short-lived positron-emitting radionuclides, *e.g*., ^18^F and ^68^Ga, further enables higher sensitivity and resolution imaging by PET compared with SPECT. This study aimed to evaluate new B7–H3-targeting Affibody molecules with the goal of improving the imaging properties of Affibody molecules used for PET imaging of B7–H3-expressing tumors. Particularly, high-imaging contrast is essential for major metastatic sites such as in liver, lung, and bone, and the small size and high affinity of Affibody molecules are critical characteristics to provide high contrast. The feasibility of preclinical SPECT imaging B7–H3-expressing tumors using an Affibody molecule labeled with ^99m^Tc was shown earlier.^31^ However, the tumor uptake was unsatisfactory, and the targeting properties of the Affibody molecules needed to be improved to obtain a higher signal from tumor and less background, resulting in an improved imaging contrast. We have shown that the affinity maturation of anti–B7-H3 Affibody molecules leads to improved imaging contrast.^32^ Moreover, the use of the residualizing label with better retention in tumor resulted an improved tumor uptake and a significantly higher tumor-to-blood ratio compared to nonresidualizing ^99m^Tc-labeled Affibody molecules.^34^ However, the binding properties of Affibody molecules are not the only factors influencing uptake in tumors and normal tissues. Coupling a radionuclide is typically done by attaching a chelator or prosthetic group to the targeting protein, which can affect the properties of the surface of the probes. This can have an influence on both on-target and off-target interactions *in vivo*, thereby impacting imaging contrast. Thus, targeting properties of Affibody molecules labeled with ^68^Ga for preclinical imaging of B7–H3-expressing tumors using PET were investigated. To enable labeling with ^68^Ga, the NOTA chelator was coupled to the Affibody molecules. This chelator provides exquisite *in vivo* stability of labels and is especially useful with Affibody molecules as these molecules can withstand the elevated temperatures required for stable chelating *via* NOTA. The melting temperatures were all above 54 °C, and importantly, the molecules demonstrated excellent refoldability, allowing renaturing of the molecules even after heating to 90 °C. The short half-life of ^68^Ga permits us to perform imaging a few hours after injection using Affibody molecules. Labeling with ^68^Ga was successfully performed with high efficiency (Table 3) generating highly stable conjugates (Table 4). Binding of ^68^Ga-labeled Affibody molecules was B7–H3-mediated *in vitro* and *in vivo* (Figures 7 and 9). The cellular processing (Figure 8) showed that the total cell-associated bound activity of ^68^Ga-labeled affinity matured Affibody molecules on SKOV-3 and BT-474 was similar and that the associated-bound activity to the cells was approximately 3-fold higher than for [^68^Ga]­Ga-Z_AC12_ at 3 h incubation. The internalization was slow, which is a typical pattern for Affibody-based probes. Biodistribution data in tumor-bearing mice 2 h after injection (Table 5) demonstrated that the blood concentration of [^68^Ga]­Ga-Z_B7–H3_2_, [^68^Ga]­Ga-Z_B7–H3_3_, and [^68^Ga]­Ga-Z_B7–H3_4_ was 2-fold higher than for the control variant, [^68^Ga]­Ga-Z_AC12_. The binding of a radiolabeled peptide to blood proteins could in principle slow its clearance and increase the background signal. Even minor changes in labeling strategy of Affibody molecules have previously been shown to have an impact on their off-target interactions (including binding to blood proteins) and, consequently, the biodistribution profile.^30^ Choosing an optimal labeling strategy could therefore help minimizing this effect.^42–44^ The tumor-to-blood ratio is a critical parameter in molecular imaging, particularly when evaluating the performance of radiolabeled imaging agents. For agents used both in diagnostics and therapy (*e.g*., radiotheranostics), a high tumor-to-blood ratio can predict therapeutic efficacy by confirming that the agent effectively localizes in the tumor with minimal off-target distribution. Thus, to improve imaging contrast, the activity of the imaging agent in the blood should be reduced and the activity in the tumor should be increased. Tumor uptake was two-to-3-fold higher for [^68^Ga]­Ga-Z_B7–H3_2_, [^68^Ga]­Ga-Z_B7–H3_3_, and [^68^Ga]­Ga-Z_B7–H3_4_ than for [^68^Ga]­Ga-Z_AC12_ (Table 6). Tumor-to-blood ratio was in the range of 24–37. The tumor-to-blood ratio for [^68^Ga]­Ga-Z_B7–H3_2_ was 1.5-fold higher than for the best reported ^99m^Tc-labeled Affibody molecules for imaging of B7–H3-expressing tumors using SPECT.^32^ Importantly, the increased absolute tumor uptake could facilitate more sensitive imaging and a shorter image acquisition time. A consequence of the rapid kinetics through renal filtration is high kidney uptake. This is mediated by a natural mechanism and caused by reabsorption of smaller proteins in the proximal tubules.^30^ For therapeutic purposes, one strategy to mitigate renal uptake was demonstrated where adding of a protein domain to increase the size of the molecule and hence avoid renal filtration allowed successful preclinical radiotherapy with a HER2-specific Affibody molecule.^49^

Hepatic uptake and hepatobiliary excretion of imaging agents can decrease the imaging contrast, reducing the detection sensitivity of hepatic and extrahepatic abdominal metastases, respectively. Several factors influence the hepatic uptake of imaging agents, such as the chemical nature of radiocatabolites and physicochemical properties of targeting agents and linkers.^45^ The liver uptake was 2.5–4-fold higher for [^68^Ga]­Ga-Z_B7–H3_2_, [^68^Ga]­Ga-Z_B7–H3_3_, and [^68^Ga]­Ga-Z_B7–H3_4_ than for [^68^Ga]­Ga-Z_AC12_. Thus, the liver uptake of the AC12 variant was lower than the uptake of the other variants. The higher accumulation of activity of the improved molecules in the tumor compared with the liver nevertheless resulted in high tumor-to-liver ratios. The highest previously reported tumor-to-liver ratio obtained using Affibody-based imaging agents was for [^99m^Tc]­Tc-SYNT179 (5.9 ± 0.8) at 4 h after injection.^32^ Using [^68^Ga]­Ga-Z_B7–H3_2_, the tumor-to-liver ratio was improved 1.3-fold (7.5 ± 0.7) already at 2 h after injection; [^68^Ga]­Ga-Z_B7–H3_2_ was clearly superior for imaging of B7–H3-expressing tumors *in vivo*. In addition, the uptake of [^68^Ga]­Ga-Z_B7–H3___2_ in B7–H3-positive SKOV-3 xenografts was significantly higher than in B7–H3-negative Ramos xenografts (Figure 11), which demonstrates that tumor accumulation *in vivo* was B7–H3-mediated. Considering clinical translation, it is important to point out that the Affibody molecule class has been demonstrating efficacy and, importantly, safety in several imaging studies, with more than 200 patients safely dosed with a HER2-targeting Affibody molecule^48^ and more than 1000 patients being dosed chronically for up to three years with therapeutic doses with high efficacy, safety, and with no clinical signs of immunogenicity.^29^ Hence, the drug class as such can be both manufactured according to GMP and safely dosed for extended periods of time, which is promising for the development of B7–H3 targeted Affibody molecules.

## 3 Conclusions

The use of an Affibody-based tracer containing a NOTA chelator at the C-terminus provides labeling of the anti–B7-H3 Affibody molecule with ^68^Ga. The newly introduced Affibody-based tracers are suitable for preclinical imaging of B7–H3-expressing tumors already 2 h after injection. [^68^Ga]­Ga-Z_B7-H3_2_ could be a promising candidate for further development aimed at clinical imaging in the future.

## 4 Methods

### 4.1 General

Gallium-68 was obtained by fractionated elution of the ^68^Ge/^68^Ga generator (Eckert and Ziegler AG, Berlin, Germany) with 0.1 M HCl. The eluate with the highest radioactivity concentration was used for labeling. The iTLC analysis was performed using CR35 BIO Plus Storage Phosphor System and AIDA image analysis software (from ElysiaRaytest, Bietigheim-Bissingen, Germany). The activity from cell and animal samples was measured using an automated γ-spectrometer equipped with a 3-in. NaI (TI) well detector (2480 Wizard, Wallac, Turku, Finland). Radioactivity for labeling and injection formulation was measured using a dose calibrator RDC-VIK-202 (COMECER Netherlands, 8501-XC, Jourse, Netherlands) equipped with an ionizing chamber.

*In vitro* cell studies were performed using the B7–H3-expressing ovarian cancer SKOV-3 and breast cancer BT-474 cell lines obtained from the American Type Culture Collection (ATCC). Ramos lymphoma cells (ATCC) were used to establish B7–H3-negative xenografts. Cells were cultured in RPMI medium (Flow Laboratories, Irvine, U.K.) supplemented with 10% of fetal bovine serum (20% of fetal bovine serum for BT-474), 2 mM of l-glutamine, 100 IU/mL of penicillin, and 100 mg/mL of streptomycin.

To determine significant differences (*p* < 0.05), data from *in vitro* studies and biodistribution were analyzed by an unpaired 2-tailed *t* test and ANOVA using GraphPad Prism (version 10.4.1, GraphPad Software), respectively.

### 4.2 Generation and Production of Novel Anti–B7-H3 Affibody Molecules

A maturation library based on work described in refs 32,47 was designed to further assess binders with increased affinity and improved properties. Phage display selection followed by characterization of a set of mature binders resulted in selection of lead B7–H3-binding Affibody molecules for further evaluation.

His_6_-tagged Z_B7–H3___2_, Z_B7–H3___3_, Z_B7–H3___4_, and Z_AC12_ were expressed in autoinducing medium (Overnight Express TB, Novagen) inoculated with precultures of *Escherichia coli* T7E2 clones carrying plasmids with sequence verified gene fragments of each B7–H3-binding Affibody molecule. Cell disruption was done by sonication, and clarified lysates were purified using 1 mL of the His GraviTrap IMAC column (Cytiva). A second purification step was performed using reverse phase chromatography (RPC) using a 3 mL Resource 15RPC column, followed by buffer exchange to PBS using PD-10 desalting columns (Cytiva).

His_6_-tagged Z_B7–H3___2_, Z_B7–H3___3_, Z_B7–H3___4_, and Z_AC12_ with TEV protease cleavage site and containing a C-terminal cysteine were expressed and purified as described in the previous section, with 1 mM DTT added to all purification buffers until RPC. Furthermore, a step for cleaving the tag prior to purification by RPC was included: the Affibody molecules were buffer exchanged to PBS and incubated overnight at 4 °C with His-tagged TEV protease in a 30:1 molar ratio of the Affibody molecule: TEV protease, and DTT was added to 2 mM final concentration. The incubated samples, supplemented with 20 mM imidazole, were applied on a 1 mL His GraviTrap IMAC column (Cytiva) equilibrated with binding buffer. TEV protease cleaved Affibody molecules were collected in the flow through, whereas the His-tagged material bound to the IMAC resin. The nontagged Affibody molecules were further purified by reverse phase chromatography (RPC) and buffer exchanged to 0.2 M NaAc, pH 6.0. Samples were conjugated with Maleimide-NOTA (CheMatech, cat. no. C101) in 4-fold molar excess at 24 °C and 450 rpm for 90 min. Incubated samples were buffer exchanged with 0.2 M NaAc, pH 6.0. The identity of each Affibody molecule was confirmed using RP-UPLC-MS analysis using an Agilent 1290 Infinity UHPLC-system equipped with a single quadrupole and AP-ESI and the analytical column Zorbax 300SB-C8 RRHD (Agilent).

### 4.3 Circular Dichroism (CD) Spectroscopy Analysis

His_6_-tagged Z_B7–H3___2_, Z_B7–H3___3_, and Z_B7–H3___4_ were diluted to 0.5 mg/mL in PBS. A CD spectrum at 250–195 nm was obtained at 20 °C. In addition, a variable temperature measurement (VTM) was performed to determine the melting temperature (Tm). In the VTM, absorbance was measured at 221 nm while the temperature was raised from 20 to 90 °C, with a temperature slope of 5 °C/min. To study the refolding ability, a new CD spectrum was obtained at 20 °C after the heating procedure. The CD measurements were performed on a Jasco J-810 spectropolarimeter (Jasco Scandinavia AB) by using a cell with an optical path length of 1 mm. Site-specific conjugation of the NOTA chelator to all variants was performed as described earlier.^34^

### 4.4 Binding of Anti–B7-H3 Affibody Molecules to Human B7–H3­(4Ig) in SPR

The binding of NOTA-conjugated Z_B7–H3_2_, Z_B7–H3_3_, Z_B7–H3_4_, and Z_AC12_ to human B7–H3­(4Ig) was studied using Surface Plasmon Resonance (Biacore 8K, Cytiva, Uppsala, Sweden). Human B7–H3­(4Ig) was diluted in 10 mM sodium acetate, pH 4.5, and immobilized to a chip (Series S Sensor Chip CM5, Cytiva) using an amine coupling kit type 2 (Cytiva). Binding to human B7–H3­(4Ig) by the Affibody molecules (diluted to 5 nM and 50 nM in HBS-EP+ buffer) was performed using the multicycle kinetic method.

### 4.5 Binding of Anti–B7-H3 Affibody Molecules to the B7–H3-Expressing Cell Line SKOV-3

His_6_-tagged Z_B7–H3_2_, Z_B7–H3_3_, Z_B7–H3_4_, and Z_AC12_ were tested in terms of binding to B7–H3-expressing SKOV-3 cells. The cells were placed in a V-bottom 96-well plate (0.2 × 10^6^ cells/well) and incubated at 4 °C for 1 h with Affibody molecules at decreasing concentrations from 500 nM (Z_B7–H3_2_, Z_B7–H3_3_, and Z_B7–H3_4_) or 1250 nM (Z_AC12_) to 21 pM (Z_B7–H3_2_, Z_B7–H3_3_, and Z_B7–H3_4_) or 52 pM (Z_AC12_). After 1× washing in PBS with 1% fetal bovine serum (FBS), binding of Affibody molecules was identified by an anti-His monoclonal antibody (4 μg/mL) and incubated at 4 °C for 1 h followed by an Alexa488 conjugated goat-antimouse IgG diluted 1:2000 and incubated at 4 °C for 1 h. After 2× washing in PBS with 1% FBS, the fluorescence intensity was measured by a multimode plate reader (Enspire). Each sample was tested in triplicates. Binding curves were plotted, and EC50 values were determined using GraphPad Prism software.

### 4.6 Radiolabeling and *In Vitro* Stability

For labeling with ^68^Ga, 40 μg of the NOTA-conjugated anti–B7-H3 Affibody molecule was mixed with 1.25 M NaAc, pH 4 (100–200 μL). A generator eluate (100–200 μL, 40–80 MBq) was added. The reaction mixture was thoroughly vortexed and incubated for 10 min at 60 °C. The radiochemical yield was evaluated using iTLC developed with 0.2 M citrate buffer, pH 2. Purification was performed using a NAP-5 column, pre-equilibrated, and eluted with 1% BSA in PBS. Radiochemical purity was analyzed as described for the radiochemical yield above. To cross-validate radio-ITLC data further, reverse phase HPLC was used as described earlier.^31^

To evaluate stability, fractions of the freshly ^68^Ga-labeled Affibody molecules (10 μL, 1 μg) were incubated with 1000-fold molar excess of EDTA for 2 h at 37 °C. Incubation was also performed in PBS as a control. The test was run in triplicates.

### 4.7 *In Vitro* Studies

Ovarian SKOV-3 and breast BT-474 cancer cell lines were used for cell studies representing B7–H3-expressing cell lines. The Ramos lymphoma cell line was used as a B7–H3-negative control. The B7–H3 expression levels were estimated to be 68,000, 45,000, and 250 receptors per cell for SKOV-3, BT-474 and Ramos, respectively.^31^ Cells were seeded in cell-culture dishes (35 mm in diameter) with a density of 10^6^ cells/dish for an *in vitro* study. A set of three dishes was used for *in vitro* studies.

The binding specificity of ^68^Ga-labeled Affibody molecules on B7–H3-expressing cells was tested using a saturation experiment performed, as described earlier.^31^

200-fold excess of the nonlabeled Affibody molecule and 10 nM of ^68^Ga-labeled Affibody molecules were used for this experiment. The radioactivity of cells was measured using an automated γ counter and the cell-bound radioactivity was calculated.

Cellular processing of ^68^Ga-labeled Affibody molecules by B7–H3-expressing cells during continuous incubation was studied by an acid-wash method.^46^ Radiolabeled Affibody molecules (10 nM) were added to the cells and incubated at 37 °C in a humidified incubator for 1, 2, and 3 h.

### 4.8 *In Vivo* Studies

Animal experiments were performed according to the national legislation for work with laboratory animals granted by the Ethical Committee for Animal Research in Uppsala (permit 5.8.18–00473/2021, approved 26 February 2021). Four mice per data point were used in the biodistribution experiments.

Biodistribution of ^68^Ga-labeled Affibody conjugates was studied in BALB/C nu/nu mice bearing B7–H3-positive SKOV-3 xenografts. To establish xenografts, SKOV-3 cells (10^7^ cells/mouse) were subcutaneously injected on the right hind leg of female BALB/c nu/nu mice. For *in vivo* specificity, B7–H3-negative Ramos cells (6 × 10^6^ cells/mouse) were subcutaneously implanted on the left hind leg of female BALB/c nu/nu mice. The experiments were performed 3 weeks after cell implantation. The average animal weight was 18.8 ± 1.2 g. The average tumor weight was 0.11 ± 0.04 and 0.41 ± 0.17 g for SKOV-3 and Ramos xenografts, respectively. The biodistribution of ^68^Ga-labeled Affibody molecules was measured 2 h after injection. Groups of 4 mice were injected with ^68^Ga-labeled Affibody molecules (0.4 nmol, 400 kBq, in 100 μL of PBS) into the tail vein. To test B7–H3-mediated accumulation of Affibody molecules, one group of animals bearing B7–H3-negative Ramos xenografts was injected with the same peptide and activity doses, and the biodistribution was measured 2 h after injection. Mice were euthanized by overdosing of anesthetic solution (20 μL of solution per gram of body weight: ketamine, 10 mg/mL; Xylazine, 1 mg/mL) following by a heart puncture and collecting blood. Organs and tissues were collected and weighed. The organ radioactivity was measured by using a γ spectrometer along with three standards and empty syringes for each animal. Uptake values for organs were calculated as the percentage-injected dose per gram of tissue (%ID/g).

To confirm biodistribution results, small animal PET/CT imaging was performed. One SKOV-3 bearing mouse was intravenously injected with ^68^Ga-labeled Affibody molecules (0.4 nmol, 2.5 MBq). To confirm *in vivo* specificity, one mouse bearing a Ramos xenograft was intravenously injected with the same peptide and activity dose. The mice were imaged at 2 h after injection using a PET/CT scanner (Mediso Medical Imaging Systems, Budapest, Hungary). The mice were euthanized by CO_2_ asphyxiation immediately before being placed in the camera. CT acquisition was performed using nanoScan PET/CT (Mediso Medical Imaging Systems Ltd., Hungary) immediately after PET acquisition using the same bed position. The PET scans were performed for 30 min, followed by CT examination at the following parameters: CT-energy peak of 50 keV, 670 A, 480 projections, 2.29 min acquisition time. The PET data were reconstructed into a static image using the Tera-Tomo three-dimensional (3D) reconstruction engine. CT raw files were reconstructed in real time using Filter Back Projection in Nucline 2.03 software (Mediso Medical Imaging Systems, Hungary). PET and CT files were fused and analyzed using Nucline 2.03 software (Mediso Medical Imaging Systems, Hungary) and were presented as maximum intensity projections (MIP) on a RGB color scale.

## Supplementary Material





## Notes

Deconvoluted ion sets from mass spectrometry of NOTA-conjugated (Figure S1) (PDF)
Affibody molecules confirming the correct molecular weight of the Affibody molecules (Table S1) (XLSX)
