# Supplementary material for: Preclinical Positron Emission Tomography Imaging of B7–H3 Expression Using Affibody Molecules Labeled with Gallium-68
Source: ACS Pharmacol Transl Sci. 2025 Sep 4;8(10):3509–22. doi: 10.1021/acsptsci.5c00320 (PMC12519266; doi:10.1021/acsptsci.5c00320)
Supplement: Supplementary file 1 [file pt5c00320_si_001.pdf]

**SUPPLEMENTARY MATERIAL**

# Preclinical Positron Emission Tomography Imaging of B7-H3 Expression Using Affibody Molecules Labelled with Gallium-68

Vladimir Tolmachev [1], Ekaterina A. Bezverkhniaia [2], Eleftherios Papalanis [1], Abdullah Mujahid Bin Muhammad [1], Anzhelika Vorobyeva [1], Elin Gunneriusson[3], Susanne Klint [3], Eva Ryer [3], Matilda Carlqvist [3], Wojciech Kazmierczak [3], Anna Orlova [2], Fredrik Y. Frejd [1,3] *, Maryam Oroujeni [1]

1 Department of Immunology, Genetics and Pathology, Uppsala University, 751 85 Uppsala, Sweden;
2 Department of Medicinal Chemistry, Uppsala University, 751 83 Uppsala, Sweden;
3 Affibody AB, 171 65 Solna, Sweden.
* Correspondence: Fredrik Y. Frejd (fredrik.frejd@igp.uu.se)

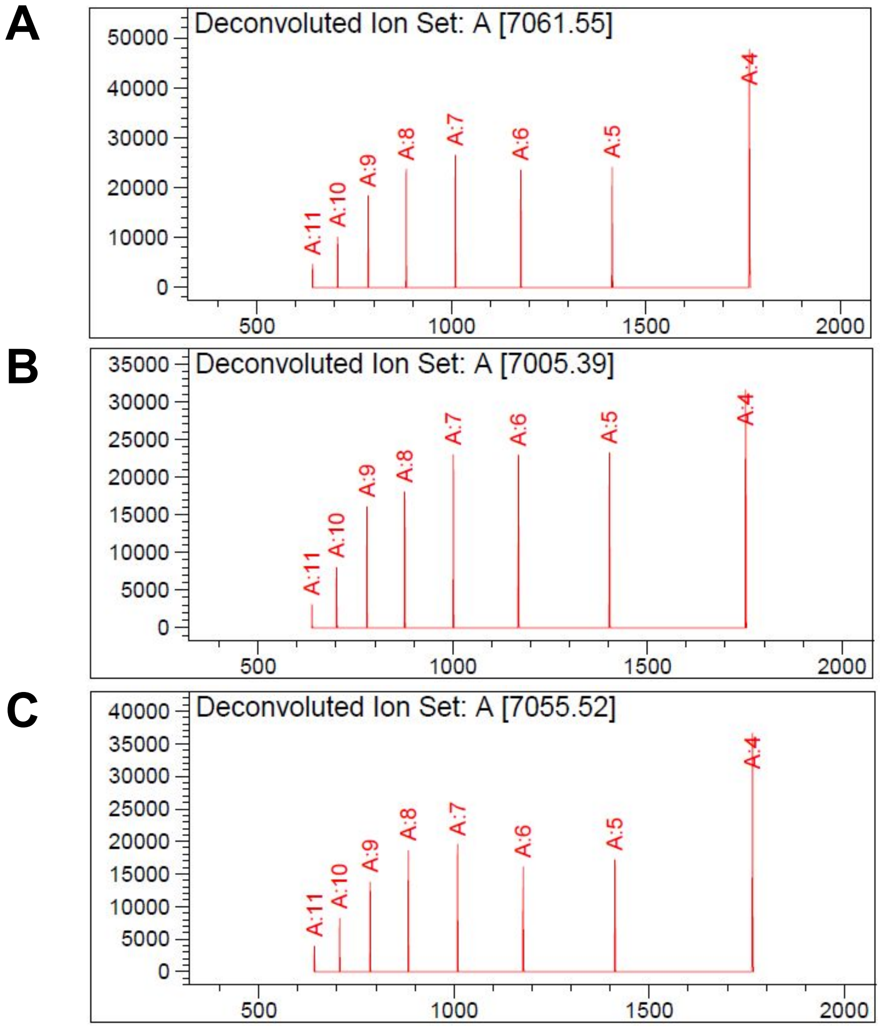

D

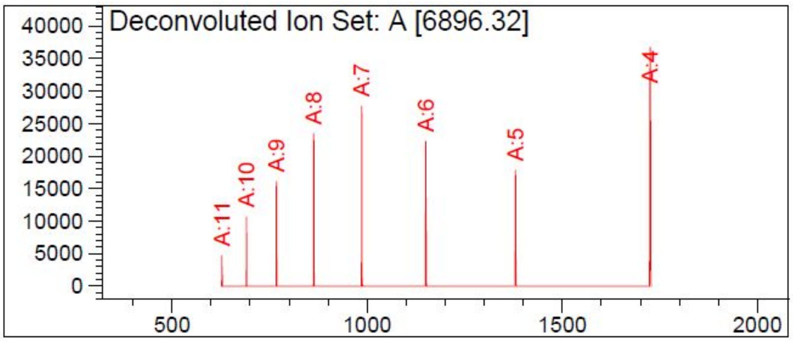


**Supplementary Figure** 1: Deconvoluted ion sets from mass spectrometry of A) NOTA-conjugated $Z_{B7\text{-}H3\_2}$, B) of NOTA-conjugated $Z_{B7\text{-}H3\_3}$, C) NOTA-conjugated $_{ZB7\text{-}H3\_4}$ and D) NOTA-conjugated $Z_{AC12}$ confirming the correct molecular weight of the Affibody molecules.
